# Perception of Electronic cigarette among young doctors

**DOI:** 10.1192/j.eurpsy.2025.985

**Published:** 2025-08-26

**Authors:** A. Hrairi, N. Rmadi, A. Kchaou, A. Chhaider, F. Dhouib, M. Hajjaji, K. Jmal Hammami

**Affiliations:** 1 Occupational medicine department, Hedi chaker university hospital, University of Sfax; 2 Family medicine department, University of Sfax, Sfax, Tunisia

## Abstract

**Introduction:**

The electronic cigarette (EC), invented in 2006, represents a recent phenomenon that is increasingly discussed especially in healthcare environment. Beliefs regarding this form of consumption are very diverse.

**Objectives:**

This study aims to assess the perception of young doctors towards EC consumption.

**Methods:**

An online survey had been conducted inspired from the French health barometer (2017). Data were collected through an anonymous questionnaire using Google Forms. The recruitment of participants was based on social media platforms combining young doctors.

**Results:**

A total of 203 young doctors had responded to the questionnaire. Most of participants were female (71%) with a mean age of 25.64 ± 2.64 years. More than half (60%) were resident doctors. Non-smokers represent 79.3% of our population. At the time of the survey, only 14 subjects (6.9%) had used EC or experienced vaping. The evaluation of perceptions with regard to vaping had showed that 30% of participants disagreed with the fact that EC consumption represents a measure to promote the cessation of tobacco use and 82% hadn’t recommended it to their patients. A proportion of 47.3% thought that vaping is as harmful as smoking and 60% agreed that e-cigarettes may contain toxic chemicals. More than half (51.6%) almost agreed that vaping may encourage ex-smokers to become nicotine addicts.

**Conclusions:**

We may conclude that EC represents for young doctors an emerging form of consumption that is not a safe or healthy alternative to smoking.

**Disclosure of Interest:**

None Declared

